# Assessing Healthcare Integration: An Integrated Palliative Care System in Spain

**DOI:** 10.5334/ijic.8613

**Published:** 2024-10-03

**Authors:** Meritxell Mondejar-Pont, Laura Rota-Musoll, Xavier Gómez-Batiste, Anna Ramon-Aribau

**Affiliations:** 1 Research Group Methodology, Methods, Models and Outcomes of Health and Social Sciences (M_3_O), Faculty of Health Sciences and Welfare, Universitat de Vic-Universitat Central de Catalunya (UVic-UCC), Vic, Spain; 2 Institute for Research and Innovation in Life Sciences and Health in Central Catalonia (IRISCC), Vic, Spain; 3 Faculty of Medicine, University of Vic-Universitat Central de Catalunya (UVic-UCC), Vic, Spain

**Keywords:** integrated care, health system, palliative care, chronic care, evaluation study, atención integrada, sistema de salud, cuidados paliativos, atención crónica, estudio de evaluación

## Abstract

**Introduction:**

This study explored the Osona palliative care system, recognized internationally for its good results in managing the chronic patient. The literature notices a gap of models that evaluate integration in healthcare systems. This study assesses the degree of integration of the Osona palliative care system, as well it implements a model that evaluates integration.

**Methods:**

This research used a qualitative methodology, involving a case study design with three study phases. The first phase involved reviewing primary sources, followed by conducting interviews. The final phase entailed comparing the findings with a theoretical model to analyse and validate the results.

**Results:**

The study found the integrative elements that the Osona system includes such as: multidisciplinary teams, leadership and a palliative care system that is cost-efficient. It also found aspects to improve including collaboration, continuity of care, early patient identification and lack of funding.

**Discussion:**

Our findings suggest that the Osona system has made significant progress toward integration, even though it continues the path of ongoing development in integrated care.

**Conclusion:**

This research found that the Osona palliative care system includes many integrating aspects such as multidisciplinary teams, leadership and the system’s cost-efficiency. Nevertheless, some aspects need changes such as continuity of care, collaboration, enhanced early patient identification and increase funding. Furthermore, this study provides an example of how to assess integration in a system.

## Abstract (es)

**Introducción:**

Este estudio investiga el sistema de cuidados paliativos de Osona, reconocido internacionalmente por sus buenos resultados en el manejo de pacientes crónicos. La literatura señala una falta de modelos que evalúen la integración en los sistemas de salud. Este estudio evalúa el grado de integración del sistema de cuidados paliativos de Osona y, además, implementa un modelo que evalúa dicha integración.

**Métodos:**

Esta investigación utilizó una metodología cualitativa, con un diseño de estudio de caso con tres fases. La primera fase consistió en revisar fuentes primarias, seguida de la realización de entrevistas. La fase final implicó comparar los hallazgos del estudio con un modelo teórico para analizar y validar los resultados.

**Resultados:**

El estudio identificó elementos integradores que incluye el sistema de Osona, como equipos multidisciplinarios, liderazgo, y un sistema de cuidados paliativos rentable. También encontró aspectos a mejorar, incluyendo la colaboración, la continuidad de la atención, la identificación temprana de pacientes y la falta de financiación.

**Discusión:**

Nuestros resultados sugieren que el sistema de Osona ha logrado un progreso significativo hacia la integración, aunque continúa en el camino de un desarrollo continuo en la atención integrada.

**Conclusión:**

Esta investigación encontró que el sistema de cuidados paliativos de Osona incluye muchos aspectos integradores, como equipos multidisciplinarios, liderazgo y eficiencia en costos del sistema. Sin embargo, algunos aspectos requieren cambios, como la continuidad de la atención, la colaboración, la mejora en la identificación temprana de pacientes y el aumento de la financiación. Este estudio aporta un ejemplo de cómo evaluar la integración en un sistema.

## Introduction

The world is experiencing a rapid increase in the number of older people, according to the World Health Organization [WHO], the number of people over 60 will double between 2015 and 2050, accounting for 22% of the total population [1]. Nonetheless, while life expectancy has increased to 73 years, a healthy prospect is only expected until 63 years of age [2]. Therefore, this ageing population currently lives about ten years more than before, but during these years they might suffer several diseases and disabilities. Today, older people are living longer, but many suffer from multimorbidity, a state of complexity that is not related to a specific disease but rather to the advanced stage of life [3].

An older age population often experiences multiple chronic diseases, hearing loss, cataracts, back and neck pain, osteoarthritis, COPD, diabetes, depression and dementia as well as what has been commonly called geriatric syndromes such as frailty, urinary incontinence, falls, delirium and pressure ulcers among others, which are not associated with a specific disease, but they are the result of the later state in life. These complex health conditions are contributors to the death of this ageing population. The prospect of death from chronic and non-communicable conditions worldwide is projected to be 69% in 2030 [4].

This growing chronically ill population presents a new challenge for healthcare systems, increasing pressure on existing care services due to their extensive use [5]. This situation results in higher costs for healthcare systems, as they need to address the increased demand for services required by an aging population [6].

In this context, it is important to consider how health systems can better respond to ageing, chronically ill, palliative, and end-of-life patients.

Historically, health systems have developed palliative care [PC] services for patients with terminal illnesses. Today, many health systems have expanded the scope of PC to include patients with chronic conditions and complex conditions [7]. As described by the WHO, PC is the care of patients with life-threatening illnesses that aims to provide chronically ill and end-of-life patients with better symptom control, a reduction in suffering, and an overall improvement in their quality of life [8].

The European Union and the WHO have supported care integration, urging the world health care systems to reorient their mission to offer Integrated care to better serves the ageing chronically ill population [9].

Integrated care is defined as the combination of multiple services across different levels of care to enhance both the quality and efficiency of health services [10]. The goal of Integrated care is to achieve the “triple aim”: better patient experience, better health results and cost-effective services [11]. This efficiency is achieved through the provision of coordinated and cost-effective services [12]. Integrated care should be applied at all levels of care: macro level [policy], meso level [organisations and professionals] and micro level [interventions and clinical practices] [13]. Integrated care models are shown to deliver better health outcomes for older patients with chronic conditions compared to traditional systems, offering a more favourable return on investment for the healthcare system [14]. Additionally, Integrated Palliative Care Systems [IPS] provide enhanced support for PC professionals, caregivers, and patients [15].

According to the literature, IPS seems to be the best response to ageing and chronically ill patients. Nevertheless, many authors argue that there is a gap in appropriate evaluation models to assess the degree of integration of health care systems. This study conducted a qualitative investigation in the Osona Palliative Care System [OPCS], which has a long history of PC and integrated care [9]. The study collected data from documentation, professionals at the organizational level of the system in leadership positions, as well as professionals at the care process and service delivery level. In addition, this study used the Integrated palliative care system essential elements: Blended model of research and practice to assess the level of integration of the system [16].

The main purpose of this study was to assess the degree of integration of the OPCS, with the following specific objectives: 1] to describe the OPCS at the level of structural organization; 2] to identify the OPCS aspects that make it integrated at the care process and service level; 3] evaluate the OPCS degree of integration at both the system organization and care process levels, and 4] implement a model that evaluates integration.

## Research Methods

### Design and Setting

This research followed a qualitative method with a constructivist research methodology using a case study approach that incorporated both primary and secondary sources of information. This qualitative method aims to comprehend a phenomenon to construct knowledge and seeks to understand the complex relationships in reality [17] by inductively developing theory or pattern meanings [18]. In our study, this translated into exploring the diverse perspectives of professionals in the OPCS.

To observe the degree of integration of the OPCS, this research used a case study approach, which allows for an in-depth analysis of a particular case, often a programme, or process [18]. After researching integrated palliative care Systems in Spain, we identified Osona as a reference place, due to its extensive experience in the field and the internationally recognized experts who have driven changes in this system for complex chronic patients [9]. Specifically, the study focused on the SISO organization [Integrated Health System in Osona], as an instrumental case to explain the OPCS in general. The study was conducted in Osona, Spain, a mixed urban-rural region [19]. The OPCS is characterized by an integrated view of patient care [9].

This study was granted approval by the Research Ethics Committee [name deleted for anonymity]. This qualitative research study followed scientific rigor criteria to ensure accuracy and trustworthiness through credibility, transferability, dependability, and validation [20]. Credibility was achieved through a year-long engagement with data collection, triangulation was performed by using different research techniques such as document review and interviews. The interviews included four different types of informants based on their professional specialties providing diverse views on the research topic. Peer debriefing was performed by the researchers, who consistently critically assessed the results. Peer debriefing involved two researchers critically assessing results. Additionally, the initial developed matrices were piloted and refined with the assistance of an external researcher. Comprehensive document searches and interviews ensured material adequacy. Finally, validation was ensured through triangulation, reflexive activities to avoid biases, and detailed theoretical and analytical descriptions.

### Participants

In this study, the initial participants were selected using purposive sampling based on the judgement of PC experts. This type of sampling is useful in gaining a deep understanding of the case under study rather than generalizing [21] and ensures that the data collected is information-rich and conducive to an in-depth understanding. This sampling method enhances the study’s ability to address its research goals by focusing on cases that can provide the most pertinent and meaningful information. The participants included eight members holding strategic and managerial positions in the work team group specialised in chronic and terminally ill patients at the SISO organization. The professionals are managers of diferent entities and organisations in different levels of care such as hospital, primary care, emergency care and nursing homes. The study then followed a stratified purposive sampling using a snowball technique to explore the views of other professionals in the service care level. Each manager was asked to refer key informants in their organisation in the areas of nursing, social work and medicine specialised in caring for PC patients. The participants in the case study totalled 24 professionals: eight managers, eight nurses, four social workers, and four doctors.

### Procedure

The study was conducted in three phases, with the researchers using document review and interviews as data collection methods [see Table 1. Methodological design].

**Table 1:** Methodological Design.


OBJECTIVES	STUDY STAGE	DATA COLLECTION	THEORETICAL PROPOSITIONS FROM LITERATURE	DATA ANALYSIS

Description of the OPCS structure/organizational level	Phase I	20 documents and databases with contextual data	Evaluative framework of integrated care [6]	Directed Content Analysis

Identification of the integrative elements that the OPCS includes and excludes at the process of care level	Phase II	24 Interviews with leaders and care delivery professionals	Scoping review study essential elements of integrated care [22]	Directed Content Analysis

Evaluate OPCS degree of integration at both the system organizational and process of care process level	Phase III	Documents and Interviews from this study	Blended model of research and practice [16]	Triangulation


In Phase I, document and databases were reviewed to find information related to the first study objective, to describe the OPCS at the organizational structural level. The documents and databases review provided contextual information about the OPCS, its structure, and its organizational characteristics. The review and analysis of the primary sources helped the researchers to achieve the first objective: to describe the structure and organization of the OPCS. Twenty sources of information were found, of which eighteen were articles, health plans, reports, and web sites and two were databases related to health care indicators and statistics [see Appendix A for the primary sources list]. To describe the OPCS, the literature was first reviewed, with the purpose of finding frameworks that could provide guidance into what elements, structures or processes needed to be assessed and described for this system. As Bainbridge et al. [2010] indicated, there are few frameworks that specifically evaluate PC systems, and so they developed their own [23]. Their framework was utilized for the assessment of the OPCS, by developing a set of question that guided the data extraction process regarding the OPCS structure, characteristics, financing, providers and organisation.

In the analysis phase following the deductive approach, primary sources were thoroughly read to find and classify data related to the main categories sought. The main categories were formulated as questions to guide the document data extraction and classification. For example, for the category OPCS structure, the question formulated was: “What is the nature of the OPCS context and structure?” Then, all the information related to the question was classified together [see Appendix B Documents categories and codes questions].

Phase II involved individual in-depth interviews with the OPCS professionals, which enabled this study to thoroughly explore and understand the participants’ views on the OPCS system structure and on the degree of integration of the system offered to patients. These semi-structured interviews took place either in person or by telephone and lasted approximately 30 to 40 minutes. These interviews were recorded and later transcribed [see Appendix C Interview, to see the interview questions].

Regarding the second study objective which aims to identify the OPCS aspects that make it integrated at the care and service level, this research based its analysis on a Scoping review study [22]. This study stablished the theoretical propositions that guided the coding process, during which researchers developed categories and codes related to the included and excluded elements in the OPCS that facilitate integration [see matrix of categories and codes generated for the interviews data analysis in Appendix D]. After analysing the interviews, we observed that the data reached a point of redundancy indicating saturation. No new codes were generated, and the existing codes were sufficiently elaborated, therefore no further interviews were conducted. Table 2 depicts the categories and codes developed for the integrated included aspects in the OPCS [see Appendix E for the excluded elements in the OPCS].

**Table 2:** Included Elements in the OPCS Categories and Codes.


PRELIMINARY CODES	GROUPS OF CODES	SUBCATEGORY	GENERIC CATEGORY	MAIN CATEGORY

Various screening tools	NECPAL others screening tools	Shared screening tool	PC standard screening tool	Standard screening tool

Identify and label PC patient		PC patient identification		

Information	Multiple information platforms	Multiple Information systems	Shared information system	Information system

		Multiple information modalities		

Various documents	Documents shared	Information shared by all		

Various professionals	Competent and qualified prof.	Qualified professionals	Professionals, resources and services	Funding and resources

Various services	Existent multiple services	Multiple services		

Communication	Professionals’ coordination	Professionals’ collaboration	PC professionals’ collaboration	Coordination

Same vision	Same vision	Professionals’ shared vision		

Patient intervention before complications	Identification/intervention	PC patient effective identification and intervention	PC patient early detection and intervention	Early intervention

24/7	24/7	24/7 care	PC system continuity of care	Continuity of care

Intra-level network	Network continuity	PC continuity		

Leaders	Leaders	Leadership	Supportive policies and leadership	Supportive policies and leadership

Dependency law	Policies	Policies		

Case manager	PC case manager identification	Case manager	PC case management	

Needs, preferences, opinions	Patient centred	PT preferences	Patient centred PC care	Patient centred

PT centred care		PT centred care		

Intermediate care	Intermediate care	PC specialised intermediate care	PC specialised intermediate care	

Motivation of professionals	Motivation of professionals	Motivated Professionals	Motivated PC professionals	

Interdisciplinary	Multidisciplinary	Multidisciplinary team	Inclusive multidisciplinary team	Multi/interdisciplinary team

Education, training	Education/training	Education/training	Education/training	Training and education

Implementation	Implementation	PC implementation	PC implementation model	Standard implementation model

Specialised care reduces cost	PC services cost	Specialised PC care	Specialised PC cost-efficiency	Cost-efficiency


Primary sources and interviews were subjected to deductive analysis as explained in the directed content analysis model [24]. Two members of the team analysed documents and interviews following the frameworks presented next, these guided the identification of categories and codes until consensus was reached. The researchers first, collected primary sources and interviews; then they developed a matrix of the main categories they were looking for, followed by the development of a coding rule system with anchor examples for each category to clarify what should be included and excluded for each category. Later, the documents and interviews were read several times by the researchers to understand the content. General categories, subcategories, and preliminary codes were then created. Finally, relationships, similarities, and differences were found within the main categories, subcategories, and generic categories, which were explained later in the results section.

**Figure 1:**
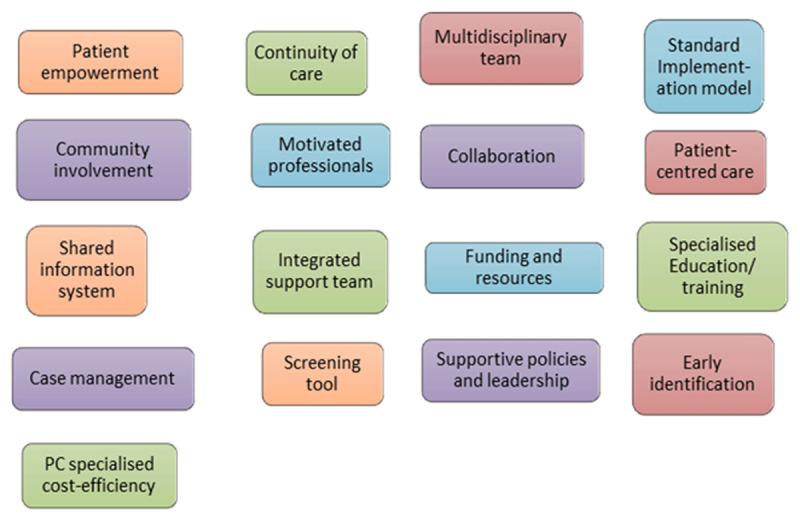
Adapted figure. Essential IPCS Elements: Blended Model of Theory and Practice.

Finally, to address the third objective of assessing the overall degree of integration of the OPCS, this study compared the data found in primary sources describing the structural organization of the system, and interviews about the care process. This comparison was made against the model found in the study about Essential integrated palliative care elements: Blended model of research and practice [16]. This blended model suggests essential elements for and integrated palliative care system by combining findings of the literature research and perspectives of practitioners working at the service level. This model was developed to fill the gap between research and practice, since as explained in many studies practitioners sometimes do not use the finding of academic research studies since it seems that often they do not reflect the practice level complexity [25,26] [see Blended model in Figure 1].

## Results

In order to respond to the study goals the results were organized within the phases in which the research was conducted. Phase I describes the structural organization in the OPCS with the results obtained from primary sources. Phase II identifies the elements that facilitate integration in this OPCS at the process of care level from the interviews. Phase III shows the results of the OPCS assessment integration level, both at the structural organizational and process of care level by comparing it to the Essential IPS elements in the Blended model of research and practice from the study previously mentioned.

### Phase I- The OPCS system at the structure organizational level

As explained in the methodology section, twenty sources of information were collected: two databases and eighteen documents. These sources of information were analysed following a PC evaluative framework [3], from which the main data collection topics were developed: environmental and contextual factors, financial situation, system characteristics, providers’ characteristics, information sharing and organizational factors.

#### Environmental and contextual factors

The Osona region is one with a medium-sized population in Catalonia, inhabited in both rural and urban areas, with a growing immigrant population. Within the region 18% of its population is 65 or older, with a larger group of women than men, many of whom are considered chronically ill patients. These chronically ill patients suffer from many conditions in two or more body systems, with a high and very high risk of admission to hospital, need for care, and death.

#### Financial situation

The system is well equipped with a general hospital, eleven primary care clinics, two intermediate care units, one mental health centre, and several nursing homes. It is in general also well equipped with health care professionals. The system also has a 24/7 vision to provide continuity of care in which exacerbations of chronic patients’ complications are covered by a planned strategy and shared individual plan. It also has a specialized service that provides 12-hour coverage to chronic and end-of-life patients requiring care.

#### System characteristics

This region has an organization called the SISO, which aims to coordinate health efforts in the area. There is, some care fragmentation, mostly between social and health areas, and sometimes caused by the after-hours care services.

The system implements a patient-centred PC, following the different Catalan health plans, with the goal of offering services that are specialized, comprehensive, accessible, and patient-centred. The chronic patient is well identified with a standard tool, the NECPAL, which is extensively used by professionals in primary care. However, as mentioned, there are some difficulties in the identification process, such as not all care levels identifying PC patients, not identifying patients early enough, not identifying all PC patients in need of this service, and in differences in the usage of the screening tool.

Regarding the standard of implementation, the 2011 Chronicity prevention and care programmes set a strategic plan to follow up with PC patients, developing specific strategies and services for the chronic patient.

#### Providers’ characteristics

Professionals in this system are competent, specialized and trained in PC, and they work cooperatively and in a multidisciplinary manner as the Catalan health plans specify. However, there is also a need for more specialized professionals, to increase the ratio of professionals per patient and for continuous specialized PC training.

#### Information sharing

The system has several platforms in which professionals share patient information and several patient documents, such as the patient’s medical history, the PIIC and PDA. The OPCS works collaboratively and with a patient-centered goal. Nevertheless, it seems there are too many online platforms on which to share patients’ information, and although the multidisciplinary teams work collaboratively, it has been suggested that teams should try to work in a more integrated manner with the diverse care levels serving patients.

#### Organizational factors

There are PC services, resources and policies, but there is a need for more specific PC policies and a greater number of services, funding and resources to fully cover the patients’ needs.

### Phase II- The OPCS as an integrated system at the process of care level

In order to find out the elements that the OPCS includes and excludes as an integrated system, the following question was posed to service level participants in the interviews: “What elements does this system include, and exclude as an integrated palliative care system?” [Table 3].

**Table 3:** Included, excluded elements and elements needing improvement in the OPCS to facilitate integration.


OPCS SERVICE CARE LEVEL VIEW		

INCLUDED ELEMENTS	ELEMENTS NEEDING IMPROVEMENT	EXCLUDED ELEMENTS

PC standard screening tool	Screening tool difficulties	Volunteer network

Shared information system	Better shared information system	

Resources, professionals and services	Missing PC funding and resources	

Professionals’ collaboration	Need to improve collaboration	

Early PC patient detection and intervention	Problems in the process of early PC patient identification and intervention	

Continuity of care in the PC system	Problems with continuity of care	

Supportive policies and leadership	PC specific policies	

PC case management	Need more PC case management	

Patient-centred care	Need more patient-centred care	

Education and training	Need standardised education and training	

PC implementation model	PC standard implementation model difficulties	

Inclusive multidisciplinary team		

PC specialised intermediate care		

Motivated PC professionals		

Specialised PC for cost-efficiency		


The included elements, or those needing improvement, in the OPCS that were mentioned were: a PC standard screening tool, which, as suggested should be improved by including all providers usage and training. A shared information system, which, as noted, should be centralized into one platform and inter-operability for all improved. Resources, professionals and services all need to be increased for PC, as well as more professional collaboration among providers. It was also stated that an increase in early PC patient detection was needed, and continuity of care in the PC system needs improving between some services and areas such as the health and social area. More supportive policies and their implementation, PC case management, education and training, a PC implementation model and patient-centred care were also mentioned. Having good leadership, PC specialized intermediate care, motivated PC professionals, inclusive multidisciplinary teams and specialized PC for cost-efficiency were all mentioned as elements that the system has and that work efficiently. The only element that was mentioned as excluded in the system was having a network of volunteers.

### Phase III- Evaluation of the OPCS integration level, both at the system structural and process of care level compared to the Essential IPS elements: Blended model of research and practice

The elements found at the structure and process of care level that facilitate integration on the OPCS were compared with the essential elements for an integrated palliative care system in the Blended model of research and practice [see Figure 1, above].

The results show that the coinciding elements are having multidisciplinary teams, a standardized implementation model, a shared information system, training and education, a standardized screening tool, care that is patient-centred, policies and leadership that support PC; all of this makes a system that is more cost-efficient.

The elements that were only mentioned as facilitators of integration, without mentioning that they needed any change or improvement in the OPCS at the structural and service care levels coinciding with the essential IPS elements blended model were: having a multidisciplinary team, leadership, and a specific PC system that was cost-efficient [See Table 4].

**Table 4:** Comparison of integration elements at the structure, process of care and Integrated elements in the Blended model.


ELEMENTS THAT FACILITATE INTEGRATION AT THE STRUCTURAL LEVEL IN THE OPCS	ELEMENTS THAT FACILITATE INTEGRATION AT THE PROCESS OF CARE LEVEL IN THE OPCS	ESSENTIAL ELEMENTS FOR AN INTEGRATED PC SYSTEM IN THE BLENDED MODEL

**Standardised implementation model**	**PC implementation model**	**Standard implementation model**

**Multidisciplinary teams**	**Inclusive multidisciplinary team and**	**Multidisciplinary team**

**Shared information system**	**Shared information system**	**Shared information system**

**Training/education**	**Education and training**	**Specialized education and training**

**Standardised screening tool**	**PC standard screening tool**	**Screening tool for identifying patients**

**Patient-centered care**	**Patient-centred care**	**Patient-centred care**

**Policies and Leadership**	**Supportive policies and leadership**	**Supportive leadership and policies**

**PC system cost-efficiency**	**Specialised PC for cost-efficiency**	**PC specialized cost-efficiency**

	Continuity of care in the PC system	Continuity of care

	Professionals’ collaboration	Collaboration

	Motivated PC professionals	Motivated professionals

	Early PC patient detection and intervention	Early PC patient detection

	PC case management	Case management

	Resources, professionals and services	Funding and Resources

	PC specialised intermediate care	Empowered patient

		Social Services

		Community involvement

		New Integrated PC support team


## Discussion

The study aimed to achieve its objectives by comparing results obtained from primary sources on the OPCS and interviews with professionals to the proposed essential elements in an IPS Blended model of research and practice [see Figure 1]. This comparison sought to identify the integrative elements within the OPCS at both the structural and process of care level, assessing their contribution to the overall system integration.

### Elements that facilitate integration at both the OPC systems’ structure and process of care level

At the structural and process of care level effective integration is present with multidisciplinary teams with competent, motivated and well-trained professionals from different disciplines. It is essential to have multidisciplinary teams to offer integrated care, since these teams are crucial to provide patients with quality care and achieve patients’ health goals [27]. Moreover, the significance of interdisciplinary teamwork ensures holistic care, since it addresses not only the patients’ physical symptoms but also their emotional, social and spiritual needs [28].

Emphasis is also placed on having a specialized PC program at both levels structure and process of care. This program uses a standardized screening tool called NECPAL for patient identification, implements a standardized intervention model, and employs a shared information system. All these aspects together, contribute to enhancing patient care in an integrated manner. The use of specialized PC programs, incorporating a standardized implementation model and a screening tool, is crucial to offer with integrated care [28]. This kind of program provides an external structure that facilitates the development of plans and care models, assisting professionals in organizing care more effectively and improving interactions between services and patients. Furthermore, these specialized programs provide with tailored care and expertise for complex patient cases [29] thereby becoming more cost-efficient models essential for sustainable health care systems [30].

At the process of care level, the significance of having experts and recognized leaders in the region is denoted as a positive aspect to the integration of the PC system. Having influential leaders in the PC sector, has not only triggered new research and innovation in the Osona region, but also it has led to the formulation and advancement of PC policies. This occurrence is reinforced by authors who explain that knowledge is developed by researchers and it is utilized by policymakers to create effective health policies [31]. Since 2011, there are policies in place that have guided PC initiatives in the region, leading to better patient management and increased cost-efficiency in the PC system. As other studies indicate policies are needed to promote systemic changes and to foster a culture change that values palliative care as an integral part of healthcare [32].

Furthermore, PC patients have been better managed in this region with primary and intermediate care and their use of acute and emergency services has decreased, leading to a more cost-efficient system [9]. As this author explains having an streamlined outpatient system that efficiently transitions patients to a more cost-effective primary care setting can reduce the duration and cost associated with inpatient stays [33].

### Elements requiring modifications to enhance integrated care both at the structure organizational and process of care level within the OPCS

Certain aspects within the Osona system promote integrated care; however, some of them need modifications at both the structural and process of care levels. Professionals’ collaboration and continuity of care between certain service levels, hospital, intermediate, primary and homecare, need to improve. As highlighted by some studies collaboration among professional is a pivotal element to palliative care integration [32]. Lack of continuity of care leads to fragmentated care. Fragmentation in the continuity of care between health and social service areas has been specially mentioned at both levels. Although there is a policy aimed to unified social and health care in this region, there is a lack of alignment with their directives [34]. While in succesful intergrated care systems, such as in England, where social and health services unified [35], challenges still persist in some settings where the integrated structure is not sufficent to provide with adequate integrated care [28]. As explained in a report on social and health care integration in this region, numerous barriers hinder the integration of health and social areas including: lack of leadership, trust, coordination, differences in their discipline culture, training, and funding [36]. Additonally, in response to the issue with continuity of care, several respondents expressed the need for improved 24/7 after-hours coverage to ensure seamless care continuity. This study also confirms the need to provide after-hours care and access to the medical team for PC patients, ensuring they can remain at home and receive continuous care [5].

Integrated care aims to address fragmentation [37] which often leads to adverse outcomes for patients [38]. Fragmentation increases the risk for co-morbidities and raises the health care cost due to higher hospital usage, unnecessary test and frequent doctors’ visits [38]. Ensuring continuity of care across hospital, intermediate, primary, and homecare services is essential for the OPCS. Therefore, it is imperative to enhance efforts to improve continuity of care to achieve better patient outcomes and reduce costs.

With regards to case management and patient-centred care, although the system includes these aspects, it seems that further development and a more homogeneous application would benefit the PC patient. Case management programs for complex patients are commonly used as an effective strategy for integrated care. Case managers facilitate stability between the health and social care teams, promote patient-centered care and enhances patients outcomes and care experiences while decreasing healthcare misuse and cost for the system [39]. It is clear further development and more consistent application of case management within the OPCS will be crucial for optimizing care delivery and achieving better results for patients.

Additionally, despite the existence of a screening tool to identifying the amount of PC patients’, detection is often insufficient, and they are not identified early enough in their disease progression. These could be explained by many reasons, first, as stated by the interviewees uncertainties persist as to when and how to use the screening tool, stressing the need for more training. Secondly, the identification process is typically carried out by primary care professionals, which can be considered a strength, as these professionals are well positioned to identify the PC patient that is mostly at home [40]. However, as another author explained a PC approach should be integrated into the training of all health care professionals to ensure that every healthcare professional has the necessary skills to effectively address all patients need [41]. Consequently, all healthcare professionals should be able to identify these complex patients. Lastly, the delayed detection of patients could also be attributed to the necessity for enhancement in the identification tool. Many screening tools used for detecting PC patients often focus on predicting death and deterioration signs, rather than in anticipating palliative care needs or in predicting patients decline [40]. Shifting towards PC systems that prioritize the identification and referral of patients based on their needs rather than prognosis, holds the potential for earlier identification of these patients [41].

An additional area identified for improvement is implementing a unified information system. Electronic patients records and information sharing platforms can assist with identifying patient’s needs, planning and monitoring care, and assessing patients’ outcomes. These shared information systems also enhance collaboration and teamwork among healthcare providers, regardless of their organization location [42]. These systems should be interoperable across all healthcare providers and it should incorporate multimodal communication methods, facilitating efficient sharing of health information [43]. The multimodal system could also play a crucial role in patient care, incorporating telehealth or telemedicine. The inclusion of these technologies with other healthcare services introduces innovative approaches to managing patients requiring alternatives to traditional in-person care that can offer them the necessary care that might otherwise be unavailable [44].

Additional resources, services and funding are required to address the needs and demands of this PCS. Adequate financial resources and supportive policies sustain integration efforts [45].

The OPCS must address critical areas to optimize care delivery and achieve better patient outcomes.

### The OPCS integration level both at the system and process of care level compared to the IPS Blended model of research and practice

Some of the elements identified in this study regarding the OPCS that contributes to integration, at the structural and service care levels, align with those outlined in the Blended model. These common elements include the presence of multidisciplinary teams, effective leadership, and a specialized PC system that enhances cost-efficiency. It is clear that these aspects are well-established and are functioning effectively to integrate care in this system. Moreover, these elements are also identified as crucial for integrated care in previous research [42,43].

On the other hand, ensuring continuity of care, addressing challenges in early patient identification, and securing funding, professionals and services were indicated as essential in the Blended model, but these were mentioned in this study as requiring change for the optimal integration of the OPCS at both levels of the system. Similarly, these aspects have been recognized as essential for achieving integrated care in other studies as well [37,46]. Future efforts in the OPCS should focus on improving continuity of care, enhancing case management, advancing early detection of patients, implementing technological improvements, and ensuring effective resources allocation. These steps are essential for developing a more effective integrated care system.

In addition, integrated care has to take place in all the different levels of a system, at the micro [clinical and service], meso [organization and professional] and macro [system] integration [47]. Therefore, integration needs to take place in the whole system [37]. In alignment with this, the Osona region demonstrates effective integration at the meso level, while areas identified for improvement are mostly at the micro level. Conversely, at the macro level, although some policies are in place, critical elements such as regulatory frameworks and having an adequate economic and social climate within the systemic domain remain inadequately defined, which is crucial aspect for overall system integration [46].

Many of the studies reviewed, suggests the need for a whole system integration across micro, meso and macro dimension, to stablish an effective integrated care system. It is crucial to make clear that the Osona system is in an ongoing path to develop an integrated care PC system.

### Implementation of the Blended model and Study limitations

Future studies should take certain considerations into account when assessing the level of integration within a system. There are different studies in which they use tools and framework aiming to assess integrated care across different countries and regions, that have obtained reliable results [48,49]. On the other hand, the Blended model used in this study to evaluate integrated care cannot be considered universal since the providers view was collected from professionals from the Osona region. It is important to notice that the concept of integrated care as a united ideal framework might be reconsidered since it is influenced by the systems’ political and economic context [37]. Consequently, this might be a limitation for the transferability of these study results, as the results of this study might be context-specific, reflecting the unique needs of Osona’s healthcare system.

This suggest that although the Blended model employed in this study, is tailored to the Osona context, other healthcare systems have the possibility to create their own blended model. This model should be adapted to their PC system and context, including all relevant stakeholders, to use it as an evaluative tool. Therefore, this study can be used as a framework to asses integrated care in a healthcare system, when adapted to their own context.

Furthermore, this model was created with the input of nurses, doctors, social workers and managerial staff, presenting a partial representation of the stakeholders involved and becoming a limitation. Patients, families and other health care providers within this system were not included. This was because the research seemed to be a novel research project in this region, focusing on describing the PC system and its integration level. A smaller sample of providers was chosen to gain their view on the system, with the intent that this study would guide a future larger-scale study, which would include patients, families and other providers. It will be important to undertake this future study as integrated care might be described differently by patients, professionals, managers and policymakers, therefore, evaluating tools should consider all their perspectives [46].

This study had other limitations, including a limited number of documents available for data collection. This might have limited the information on some descriptive aspects of the system, as only the accessible shared and published documentation was included, but it is very probable that more internal reports and documents describing the OPCS were not accessible to the researchers.

## Conclusions

Currently, the ageing population encounters chronic diseases, frailty, and a longer life span. The purpose of this research was to further study the integrated response by the health care system in the context of Osona, Spain. It also aimed to assess the OPCS integration level, with the essential IPS elements Blended model of research and practice.

This research was able to describe in detail the elements that facilitate integration in the OPCS from both a structural organizational and process of care level. In conclusion, the Osona region has many elements that facilitate integrated care such as having multidisciplinary teams, innovative leaders and a specific PC system that is cost-efficient. While certain aspects exist in the system, there is a recognized need for improvement such as: having continuity of care, early patient identification and increased funding, professionals and services. Osona is on a path toward ongoing development on integrated care.

This study not only adds to the existing body of knowledge but also suggests avenues for further research and practical applications, emphasizing the value of qualitative approaches in capturing the complexity in integrated palliative care.

## Additional File

The additional file for this article can be found as follows:

10.5334/ijic.8613.s1 Appendices. Appendix A to E.
