# Supplementary material for: Assessing Healthcare Integration: An Integrated Palliative Care System in Spain
Source: Int J Integr Care. 2024 Oct 3;24(4):1. doi: 10.5334/ijic.8613 (PMC11451549; doi:10.5334/ijic.8613)
Supplement: Appendices. — Appendix A to E. [file ijic-24-4-8613-s1.pdf]

APPENDICES

Appendix A. *Documents and Databases List*

**Documents**	
1	Amil, P., Constante, C., González Mestre, A., Sarquella, E., Ledesma, A., Cabezas, C., Zara, C., Contel, J. C., Puigdollers, M., & Blay, C. (2016). Chronic and integrated care in Catalonia. *International Journal of Integrated Care*, *15*(2). https://doi.org/10.5334/ijic.2205
2	Blay Pueyo, C. (2017). *Característiques i consum de recursos sanitaris de persones amb malalties cròniques avançades: Un estudi de cohort*. http://hdl.handle.net/10803/404680
3	Consorci Hospitalari de Vic. (2019). *Consorci Hospitalary Vic. Memòria 2018*. http://www.chv.cat/consorci/comunicacio/memories/2018-memoria/professionals/
4	Generalitat de Catalunya.Departament Salut. (2012a). *Pla de Salut 2011-2015 del Programa de prevencció i atenció a la cronicitat de la Generalitat de Catalunya*. http://projectes.camfic.cat/CAMFiC/Seccions/Formacio/Docs/Material_IX_Directius/03ChronicJContel.pdf
5	Generalitat de Catalunya. Departament de Salut. (2017a). *Regió Sanitària Catalunya Central Memòria 2017*. http://catsalut.gencat.cat/ca/coneix-catsalut/catsalut-territori/catalunya-central/
6	Generalitat de Catalunya. Departament de Salut. (2012b). *El Programa de prevenció i atenció a la cronicitat*. http://salutweb.gencat.cat/web/.content/home/ambits_tematics/linies_dactuacio/model_assistencial/atencio_al_malalt_cronic/destacats_a_la_dreta/avaluacio_cronicitat_2011_2014.pdf
7	Generalitat de Catalunya. Departament de Salut. (2017c). *Recursos Específicos de Cuidados Paliativos. DIRCAT-CP 2017. Generalitat de Catalunya. Department de Salut.* http://canalsalut.gencat.cat/web/.content/home_canal_salut/professionals/temes_de_salut/atencio_al_final_de_la_vida/documents_prof/paliatiusdefinitiu.pdf%0Ahttp://salutweb.gencat.cat
8	Generalitat de Catalunya. (2019c). *Afiliació al règim general de la Seguretat Social, mineria del carbó i RETA*. Observatori Del Treball i Model Productiu. http://observatoritreball.gencat.cat/ca/ambits_tematics/mercat_de_treball/afiliacio_ss/mineria_carbo_i_regim_especial_autonoms/
9	Generalitat de Catalunya.(2016b). *Pla de Salut de Catalunya 2016-2020. Generalitat de Catalunya. Departament de Salut*. 1–161. https://doi.org/10.1017/CBO9781107415324.004
10	Gómez-Batiste, X., Martínez-Muñoz, M., Blay, C., Amblàs, J., Vila, L., Costa, X., Villanueva, A., Espaulella, J., Espinosa, J., Figuerola, M., & Constante, C. (2012b). Identifying patients with chronic conditions in need of palliative care in the general population: Development of the NECPAL tool and preliminary prevalence rates in Catalonia. *BMJ Supportive and Palliative Care*, *3*(3), 300–308. https://doi.org/10.1136/bmjspcare-2012-000211
11	Gómez-Batiste, X., Martínez-Muñoz, M., Blay, C., Amblàs, J., Vila, L., Costa, X., Espaulella, J., Espinosa, J., Constante, C., & Mitchell, G. K. (2014b). Prevalence and characteristics of patients with advanced chronic conditions in need of palliative care in the general population: A cross-sectional study. *Palliative Medicine*, *28*(4), 302–311. https://doi.org/10.1177/0269216313518266
12	Gómez-Batiste, X., Blay, C., Martínez-Muñoz, M., Lasmarías, C., Vila, L., Espinosa, J., Costa, X., Sánchez-Ferrin, P., Bullich, I., Constante, C., & Kelley, E. (2016). The Catalonia WHO Demonstration Project of Palliative Care: Results at 25 Years (1990–2015). *Journal of Pain and Symptom Management*, *52*(1), 92–99. https://doi.org/10.1016/j.jpainsymman.2015.11.029
13	Fundació Hospital de la Santa Creu. (2018). Retrieved July 20, 2019, from http://www.chv.cat/consorci/comunicacio/memories/2018-memoria-fhsc/aixi-som/les- persones/
14	Roca, J., & Ramón, I. (2010). Atención sanitaria integrada en Osona como respuesta al desafío de la cronicidad. In *Fundación Signo* (Vol. 11). https://www.fundacionsigno.com/archivos/publicaciones/2010m 9 p135.pdf
15	Observatori Socioeconomic D'Osona. (2018). Retrieved July 17, 2019, from http://www.observatorisocioeconomicosona.cat/
16	Santaeugènia Gonzàlez, S. J. (2017) Atenció Integrada i Cronicitat PPAC-PIAISS.
17	Sistema Integrat de Salut d'Osona. (2016). Sistema Integrat de Salut d'Osona Actuacions 2015.
18	Termcat. Generalitat de Catalunya. (2013). Terminologia de la cronicitat. Termcat/Centre de Terminologia. https://www.termcat.cat/ca/recursos/productes- multimedia/terminologia-la-cronicitat
Data Bases	
19	Generalitat de Catalunya. MSIQ. Mòduls de seguiment d'indicadors de qualitat (2019). Retrieved July 23, 2019, from https://msiq.catsalut.cat/index.html
20	Generalitat de Catalunya. Institut d'estadística de Catalunya. Idescat (2020.). Retrieved July 15, 2019 from: https://www.idescat.cat/?lang=en

Appendix B. *Documents, categories and codes*

Data Collection Questions	Main Categories	Generic Categories	Preliminary codes
What is the nature of the OPCS context and structure?	Density	Medium density Region	-Population<br>-Area<br>-Inhabitants
	Demography	Demography characteristics	-Age distribution<br>-Ageing<br>-Immigration<br>-Income
		Incidence of diseases in population	-Population distribution in Primary Care centres<br>-Mortality, morbidity, prevalence
What are the system's characteristics?	Services offered	Type of health centres and PC services	- Levels of care centres and services<br>-PC specific services
	Membership size	Number of services used and patients served	- PC patients served<br>- Services used
	Extent of participation in network	-Coordinating organisation	- SISO pluridisciplinary groups
	Networks ideals	Patient-centred Integral services	- Continuity of care<br>- Patient-centred care
	Policies	Health plans and programmes and services to the chronically ill	-Chronicity programmes<br>-Health plans for integral services<br>-PC directory
How is the economic and financial situation?	Network resources	Number of hospitals, clinics, centres and professionals	-Hospitals, clinics, centres<br>-Number of professionals in the health sector
	Presence of 24/7 care	24/7 vision	- Intervention plan and medical History online documents<br>- 12 hours PADES
What are the providers' characteristics?	Cooperation	Cooperation at different levels of care and services	-Collaboration
	Perceived Interdependence	Case management and multidisciplinary teams	-multidisciplinary, interdisciplinary work<br>- case management
	Specialty training	Training strategies	- Training and education
How is the information shared?	Information systems and materials	Digital health related information sharing	- Clinical History<br>- Personal health channel<br>-Si-Siso informatics system
	Standard assessment monitoring patient needs	Patient early identification	- Patient identification<br>- Screening Tools
What are the organisational factors?	Care team composition	Care team professionals	Professionals in hospitals and primary care
	Standard implementation models	Standard models	-Chronicity Programme development<br>-Care pathways
	Programme evaluation	Cost-efficiency	- Emergency hospitalisations and admissions reduction

Appendix C. *Interview*

MACA patients with advanced chronic disease
SPCO Palliative System of the Region of Osona

1. Imagine a perfect or ideal model of an integrated palliative care system that is for MACA patients [advanced chronic patients]. What elements should it have?

Please specify, within this perfect / ideal model of an integrated palliative care system for MACA patients, what these factors would be / how would they function? [if not stated above]:
- **professionals** providing palliative services in an integrated system.
- collaboration within the system of an integrated system.
- **sharing information** within an integrated system.
- **patient detection**
- organization in the system as an integrated system.
- the **services** offered to palliative patients

2. If you think about these elements for an ideal/perfect integrated palliative care system:
2.1. Please tell me which of them would be the **most important** elements, from the most to least important? And why?

3. Now I would like you to think about the SPCO. Comparing the SPCO with an Ideal Integrated Palliative Care System, what factors/elements <u>does the SPCO have</u> as an integrated palliative care system for the MACA patient?

3.1. What factors / elements mentioned above are missing or lacking in the SPCO? Why don't we have them?

4. Please tell me, from the factors / elements mentioned, which would be the most important to have [from the most to least important] given the context of the system in the Osona region [due to its geography, population, culture]

5. In your professional practice, what kind of ethical dilemmas [complicated situations as a professional] do you find yourself in when providing integrated services to the palliative patient with advanced chronic disease?

Appendix D. *Interviews, categories and codes*

- ***Multidisciplinary /Interdisciplinary teams,*** include professionals from different specialties, and disciplines such a doctors, specialists, nurses, psychologists, chaplains, physiotherapists and nutritionists. Teams organized from the inpatient to primary care and within organizations with a common goal to meet the patients' needs.

    ***-Coding rules: organization of professionals** from different specialties, disciplines, levels of care and organizations. The process of being organized in a team.*

    -Anchor sample: *"Teams that include all the professionals involved, such as a doctor, nurse and social worker".*

- ***Case management*** provides comprehensive care for the patient's physical, functional, pharmacological, social, emotional and financial needs that will interact with patients through care transitions.

    -***Coding rules:*** *comprehensive care for all patient dimensions that interacts with patients through care transitions.*

    - Anchor sample: *"A liaison nurse that works with the all the other services".*

- ***Coordination,*** meaning collaboration of services and organizations between primary and secondary services, home services from different specialties and across professionals.

    -***Coding rules: collaboration*** *between professionals, services, specialties and organizations. The* process of working together.

    -Anchor sample: *"The process of collaboration among the professionals from all the services".*

- ***Continuity of care*** can be achieved with the integration of outpatient and inpatient services provided by health systems by bridging across settings. It is provided between all agents involved in the IPC services and all the health care settings. The continuity process occurs when there is sharing of patients' documentation electronically, by care conferences, and with electronic, phone and in-person communication within the team members.

    -***Coding rules:*** *Services integration, bridging of levels of inpatient care and outpatient services.*

    -***Anchor sample****: "The connectivity of care between the different levels of care that work as a network".*

- ***Early Intervention*** **of IPC** with patients with advanced chronic conditions in need of this care. The identification of these patients should be early on in their disease process by performing early geriatric evaluations and by applying it right at the beginning of diagnosis.

    -Coding rules*: Identify patient early on in their disease [by labelling him a palliative care patient [terminal/ advanced chronic] diagnosis.*

    -Anchor sample: *"Detect the MACA patient with the first signs".*

- ***Patient-centred*** means that IPC focuses the care on fulfilling the needs and preferences of patients, their families and caregivers.

    -Coding rules*: care focuses on the patients' and their families' needs and preferences.*

    -Anchor sample*: "Care that responds to the patients' preferences".*

- ***Training and education*** involve the provision of educational opportunities to promote further learning. Training and education are necessary for the IPC provider in order to give them confidence in service provision and to develop their competence.

    -Coding rules: *develop competencies.*

    -Anchor sample: *"To educate and train these professionals in the PC model".*

- **A standard implementation model** should be applied in IPC direct services and health practices. A standard model is a key element for effective care delivery and is developed by all stakeholders.

    -Coding rules: *a standard model is implemented in all care practices by all the professionals. The same model is applied everywhere by all the professionals.*

    -Anchor sample*: "Basic model of care that in which all professionals are trained and that is agreed, explained, repeated and trained by all the professionals, a way of doing, a culture".*

- **Supportive Policies and leadership:** formal and informal guidelines, agreements, rules that guide and support the programme. Specific policies for IPC are necessary and they need to be established at the national and regional level. Leaders that inspire and encourage an optimal process for the organization's proper functioning.

  -***Coding rules****: supportive guidelines, rules or professionals that are positive for the programme.*

  -Anchor sample*: "Policies, territorial governance", "leadership", "good leaders that are concerned about PC and provide the other professionals with a lot of feedback".*

- **Shared Information Systems:** structure that facilitates the exchange of information via technology sharing [electronic health records, videoconferencing] or written/visual aids. This system facilitates the collection, reporting and availability of patient information to care providers and it insures effective IPC care delivery.

  -***Coding rules:*** *system that facilitates exchange of patients' information by all professionals [videoconference, electronic records sharing, written material].*

  -***Anchor sample****: "An information sharing system that allows for interconnectivity".*

- ***A standard screening tool*** helps providers to identify the IPC patient and determine prognosis. It screens for symptoms and assesses the patient and generates service referrals.

  -Coding rules: tool to identify IPC patient and produce care referral.

  -Anchor sample: *"A screening system that allows us to detect the population that will most need PC".*

- ***Funding* for IPC** financial resources is essential at the country and regional level. Deficient funding is an inhibitor for IPC and its proper functioning. *Resources*, both human and social, are essential for the effectiveness of an IPS.

  -Coding rules*: finances, human and social resources provided to the IPC system.*

  -Anchor sample: *"To have sedation kits", "more professionals", "more services like rehabilitation".*

- ***Cost-efficiency*** can be the result of the implementation of an IPS, since it decreases emergency visits and inappropriate admissions, reduces the time in hospitalization stays, reduces infection acquisition and delirium/confusion development from hospitalizations. It also reduces the cost of care in general for the IPC patient.

  -***Coding rules:*** *reduces IPC cost for care. Could result from IPC.*

  -***Anchor sample****: "It reduces over diagnosis and overtreatment".*

Appendix E. *Excluded Elements in the OPCS, Categories and Codes*

Preliminary codes	Groups of Codes	Subcategory	Generic Category	Main Category
24/7	24/7	24/7 care	PC care fragmentation	Continuity of care
No service continuity	Services level no continuity	Intra-level fragmentation		
Health and social services disconnection	Health and social services no continuity	Health and social service fragmentation		
Various professionals	Professionals	Professionals missing	Missing specific PC funding and resources	Funding and resources
Various services	Services	Services missing		
Various resources	Resources	Resources missing		
Funding	PC funding	PC specific funding missing		
Information platforms	Information platforms	Improve single information sys.	Shared single information system	Information system
Modalities: phone, videoconference, etc.	Information modalities	Multiple information modalities		
Communication Coordination	Professionals' collaboration	Professionals' collaboration	Improve collaboration	Coordination
Health and social services		Coordination health and social services		
Education, training	Education/training	PC specialised education and training	Need for PC standardised education/training	Training and education
Identification, labelling PC patient	Labelling, identifying PC patient screening tool	PC PT labelling difficulty	Screening tool difficulties	Standard screening tool
Routes, vision, goals for PC patient	PC standard model Implementation difficulties	PC standard implementation difficulties	PC standard implementation model difficulties	Standard implementation model
Infra-identification	PC patient infra-identification and late intervention	PC patient infra-identification and late intervention	PC patient early identification and intervention difficulties	Early intervention
Needs, preferences, opinions	Patient centred	PC patient centred	Patient centred PC care	Patient centred
Policies	Policies	Specific PC policies	Specific PC policies	Policies and leadership
Case manager	PC case manager identification	PC case manager identification	PC Case management	
Volunteers		Volunteers	PC Volunteers	
